# Power of association tests in the presence of multiple causal variants

**DOI:** 10.1186/1753-6561-5-S9-S63

**Published:** 2011-11-29

**Authors:** Yanming Di, Gu Mi, Luna Sun, Rongrong Dong, Hong Zhu, Lili Peng

**Affiliations:** 1 Department of Statistics, Oregon State University, 44 Kidder Hall, Corvallis, OR 97331, USA

## Abstract

We show that the statistical power of a single single-nucleotide polymorphism (SNP) score test for genetic association reflects the cumulative effect of all causal SNPs that are correlated with the test SNP. Statistical significance of a score test can sometimes be explained by the collective effect of weak correlations between the test SNP and multiple causal SNPs. In a finite population, weak but significant correlations between the test SNP and the causal SNPs can arise by chance alone. As a consequence, when a single-SNP score test shows significance, the causal SNPs contributing to the power of the test are not necessarily located near the test SNP, nor do they have to be in linkage disequilibrium with the test SNP. These findings are confirmed with the Genetic Analysis Workshop 17 mini-exome data. The findings of this study highlight the often overlooked importance of long-range and weak linkage disequilibrium in genetic association studies.

## Background

In a typical genome-wide association study, single single-nucleotide polymorphism (SNP) association tests, such as score tests [1,2], are used to scan the genome for possible genotype-phenotype associations. When an association test shows significance, it is commonly expected that the detected association is due to either the direct genetic effect of the test SNP or linkage disequilibrium (LD) with causal SNPs located at nearby genomic positions [3].

In this study, we show that the causal SNPs contributing to the power of a single-SNP score test are not necessarily located near the test SNP, nor do they have to be in genuine LD with the test SNP. We term this phenomenon the hyper-LD effect. The hyper-LD effect is a consequence of weak correlations between the test SNP and multiple causal SNPs. Score tests performed at rare SNPs are particularly prone to this hyper-LD effect. In this study we highlight the often overlooked importance of weak correlations between distant SNPs in association studies.

## Methods

We first derive formulas for computing the power of the score tests in the presence of multiple causal SNPs. We then give theoretical explanations of the hyper-LD effect. We focus our discussion on quantitative trait models, but, by using an argument similar to that in Appendix 1 of Chapman et al. [4], our results can be viewed as a reasonable approximation under logistic regression models for binary traits. For clarity, in this article, the term *correlation* refers to sample correlation in a finite population, and the term *LD* refers to the expected value of the sample correlation between alleles at different SNPs.

### Quantitative trait model

We consider a quantitative trait model with *J* diallelic causal SNPs at positions indexed by *j* = 1, …, *J*:(1)

where *Y* is the vector of trait values; *μ* is a constant vector of baseline mean trait values; vectors *Z_k_*, *k* = 1, …, *K*, represent measured covariates, such as age, sex, and an indicator of whether an individual is a smoker; the coefficients *α_k_* represent the effects of the covariates on trait values; the *X_j_*, *j* = 1, …, *J*, are vectors of genotypes; and the coefficients *β_j_* represent the allele effect sizes. To model an additive genetic effect, the genotypes are coded as 0, 1, or 2 according to the number of minor alleles present. Furthermore, we assume that there are no gene-gene or gene-environment interactions and that all individuals in the study are truly unrelated.

### Score tests of genetic association

Let , , and so on be the vectors of fitted values of regressing the corresponding vectors on measured covariates *Z_k_*, *k* = 1, …, *K*. The score statistic *u*[1,2] for testing association between the trait value and the genotype at a single test SNP *τ* is:(2)

Under the null hypothesis of no association between the trait values and the test SNP *τ*, the variance of *u* is estimated by:(3)

where *Z* = (**1**, *Z*_1_, …, *Z_K_*) and *s_YY_* is the sample variance of the residual trait values (**1** is a vector of ones). The score statistic *u* measures the covariance between the genotype vector *X_τ_* and the trait value vector *Y* after adjusting for measured covariates. If a covariate *Z_k_* is correlated with *X_τ_*, then the covariance between *X_τ_* and *Y* will decrease after adjusting for *Z_k_*. The effect of covariate adjustment is also reflected in the variance estimate *v* (see Section 6.3.2 of Bickel and Doksum [5] for more details). To evaluate the statistical evidence for genetic association, *u*^2^/*v* is compared to a chi-square distribution with 1 degree of freedom.

### Power of score tests in the presence of multiple causal SNPs

If there are one or more causal variants, as in the trait model given by Eq. (1), *u*^2^/*v* has a noncentral chi-square distribution with 1 degree of freedom. At each test SNP, the power (rejection probability) of the test is determined by the noncentrality parameter *λ*:(4)

Under the trait model given by Eq. (1),(5)

and the noncentrality parameter takes an intuitive form:(6)

where *N* is the number of individuals in the sample,(7)(8)

and *s_j_*_1,_*_j_*_2_ is defined as:(9)

*r_τj_* is the correlation coefficient between (the genotype vectors at) the test SNP *τ* and the causal SNP *j*, adjusted for the measured covariates. We refer to *h_j_* as the direct effect of SNP *j*;  measures the proportion of the residual trait variance explained by the causal SNP *j*. The term  reflects the cumulative effect of all causal SNPs. Equation (6) extends a corresponding equation in Clayton et al. [2] to the case of multiple causal SNPs. Equation (6) can be extended to tests based on collapsing rare variants at multiple SNPs [6,7] by letting *X_τ_* be the sum or weighted sum of the genotype vectors at the collapsed SNPs.

### Hyper-LD effect

With the help of Eq. (6), we can now summarize the theoretical findings of this study:

1. The power of a single-SNP score test reflects the cumulative effect  of all causal SNPs that are correlated (*r_τj_* ≠ 0) with the test SNP.

2. In the presence of multiple causal SNPs, individually weak correlations (e.g., *r_τj_* = 0.1) between the test SNP and the causal SNPs can collectively give rise to significant power in a score test. For example, 10 causal SNPs, each having a direct genetic effect *h_j_* = *h* and a correlation coefficient *r_τj_* = 0.1 with the test SNP would result in a noncentrality parameter of (*N* − 1)*h*^2^, which is not 10 but 100 times greater than what it would be if there were only a single causal SNP with the same effect and correlation.

3. In a finite population, observed correlations between the test SNP and the causal SNPs can be due to either LD or random fluctuations. Between SNPs in complete LD, *r_τj_* = 1. Between common SNPs that are in linkage equilibrium, *r_τj_* has an approximate normal distribution with mean 0 and variance 1/*N*[5]:(10)

This implies that even between SNPs in linkage equilibrium, about 1% of the *r_τj_* values will be greater than 2.33/*N*^1/2^ (= 0.088 when *N* = 697) by chance. In a genome-wide association study, 1% corresponds to a large number of SNPs.

4. When either the causal SNP or the test SNP is a rare SNP (e.g., when less than 30 copies of the minor allele are present in the study population), the correlation coefficient *r_τj_* will have a positively skewed distribution and thus will be more prone to random fluctuations.

5. Between a pair of a common SNP_1_ and a rare SNP_2_ with the same allele effect size (*β*_1_ = *β*_2_), the common SNP will contribute more to the power of the score test performed at the rare SNP than vice versa (*r*_21_*h*_1_ >*r*_12_*h*_2_): the correlation coefficient is symmetric (*r*_12_ = *r*_21_), but the common SNP can explain more trait variation (*h*_1_ >*h*_2_).

Findings 1 to 3 explain the causes of the hyper-LD effect: the phenomenon where the statistical significance of a score test can be explained by the collective effect of weak correlations between the test SNP and distant causal SNPs. Findings 4 and 5 explain why score tests performed at rare SNPs are particularly prone to the hyper-LD effect.

## Results

In this section, we confirm our theoretical findings with the Genetic Analysis Workshop 17 (GAW17) mini-exome data. We performed a power analysis and examined the correlations between SNPs in the GAW17 data. We focused exclusively on the unrelated individuals data set, which consists of 697 individuals from seven populations and their genotypes and phenotypes. At each of the 24,487 SNPs, we used the software GenABEL [8,9] to test the null hypothesis of Hardy-Weinberg equilibrium in each population separately. We removed 1,730 SNPs that yielded a *p*-value smaller than 10^−4^ in any of the populations, leaving 22,757 SNPs for our analyses.

### Power of score tests

The power analysis was performed using the quantitative risk factor Q1. The true simulation model [10] was known to our group. In the power analysis, the factors Age, Sex, Smoke, and Population were considered as covariates (the *Z_k_* in Eq. (1)).

**Figure 1:**
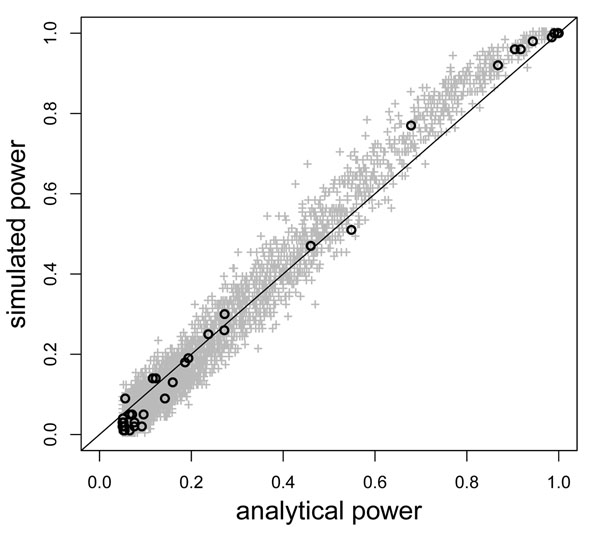
**Analytical and simulated power of score tests performed on the GAW17 data set**. Each plus sign corresponds to one SNP in the GAW17 data. The black circles correspond to the 39 known causal SNPs.

Figure 1 compares the analytical and simulated power of the score tests at the 22,757 SNPs. Analytical power was computed based on the noncentrality parameter in Eq. (6), with the *β_j_* taken to be the true values in the simulation model [10]. We estimated simulated power using the average rejection rates after tests were repeated using the first 100 replicate trait data sets. We chose a nominal level of *α* = 0.05 so that the simulated power was strictly between 0 and 1 at most SNPs. The analytical power explains 97.0% of the variance in the simulated power. The strong agreement between the analytical power and the simulated power confirms our theoretical finding 1: The power of a score test can be explained by the cumulative effect of all SNPs that are correlated with the test SNP. Note that the direct genetic effects of the test SNPs cannot explain the simulated power: Among the top 100 SNPs with the highest simulated power, only 7 are true causal SNPs; the other 93 do not have direct genetic effects on the trait values.

We next present a concrete instance of the hyper-LD effect. In this instance, none of the causal SNPs that contribute to the power of the score test are located near the test SNP. The score tests show high power (simulated power = 1) at a cluster of SNPs (C12S704, …, C12S709) on chromosome 12. These SNPs are not causal SNPs in the simulation model. In fact, none of the causal SNPs are located on chromosome 12. Note that the power of the score tests at this cluster of SNPs is still well explained by Eq. (6). For instance, 13 causal SNPs have correlations *r_τj_* > 0.1 with SNP C12S706. At SNP C12S706, the cumulative effect of the causal SNPs resulting from correlations is . This effect is greater than the direct effect *h_j_* of all but one causal SNP (the one exception being SNP C13S522 with *h_j_* = 0.23). The power of the score test reflects this cumulative effect. The fact that single-SNP score tests can show high power at SNPs not located near any causal SNP makes single-SNP tests unreliable as a tool for mapping trait genes.

### Correlations between SNP genotypes

**Figure 2:**
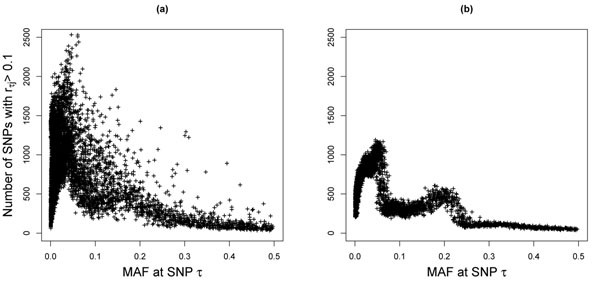
**Significant correlations between SNPs.** Significant correlations (*r_τj_* > 0.1) between SNPs in (a) the GAW17 data set and (b) a simulated data set in which all SNPs are in linkage equilibrium. On the *x*-axes is the minor allele frequency at a SNP *τ*. On the *y*-axes is the number of SNPs *j* with *r_τj_* > 0.1.

We explained (theoretical finding 2) that in the presence of multiple causal SNPs, even small correlations between the test SNP and causal SNPs can be significant to the power of a single-SNP score test. For each SNP *τ* in the GAW17 data, we counted the number of SNPs *j* having *r_τj_* > 0.1 with *τ* and plotted this number against the minor allele frequency (MAF) at SNP *τ* in Figure 2a. We did not include correlations between SNPs that are within 10 Mb of each other in the counts. Figure 2a shows that correlations of this level are prevalent in the human genome, even among distant SNPs.

To demonstrate that weak but significant correlations between SNPs can arise by chance alone, we simulated another set of 22,757 SNPs that are in linkage equilibrium with each other: The SNP genotypes were simulated to be independent and in Hardy-Weinberg equilibrium, with MAFs matching those of the SNPs in the GAW17 data. Again, for each simulated SNP, we counted the number of SNPs having correlation coefficients greater than 0.1 with it. The results are shown in Figure 2b. Figure 2b confirms that modest correlations between SNPs can arise by chance and that rarer SNPs are more prone to the effects of random fluctuations. The overall correlation level in the GAW17 data set is higher than that in the simulated data set. This is expected because genuine LD does exist in the GAW17 data.

For each SNP in the GAW17 data and in the simulated data, we also counted how many of the 39 causal SNPs in the GAW17 data had correlation coefficients *r_τj_* > 0.1 with it. The results are summarized in Table 1. The results show that a large number of SNPs can be correlated at level *r_τj_* > 0.1 with one or more causal SNPs as a result of weak LD or simply by chance. For example, 8,435 independently simulated SNPs have correlations *r_τj_* > 0.1 with at least one of the causal SNPs just by chance.

**Table 1:** Distributions of the number of causal SNPs significantly correlated (*r_τ__j_* > 0.1) with each SNP

Data set	Number of correlated (*r_τj_* > 0.1) causal SNPs								
	
	0	1	2	3	4	5	6	7	>7
GAW17	13,288	5,153	2,402	870	424	278	136	85	121
Simulated data	14,322	5,229	2,092	729	245	104	29	7	0

For each SNP in the GAW17 data and in the simulated data, we count how many of the 39 causal SNPs in the GAW17 data have correlation coefficients *r_τj_* > 0.1 with it. We find that 9,469 SNPs in the GAW17 data and 8,435 SNPs in the simulated data are correlated (*r_τj_* > 0.1) with at least one causal SNP. Note that in the simulated data set, all SNPs are simulated to be in linkage equilibrium with each other and with the 39 causal SNPs, so all observed correlations are due to chance alone.

## Discussion and conclusions

We have demonstrated a phenomenon that we call the hyper-LD effect in which the statistical significance of a score test can be explained by the collective effect of weak correlations between the test SNP and distant causal SNPs. Tests performed at rare SNPs are particularly prone to this hyper-LD effect. In the presence of multiple causal SNPs, the results of single-SNP score tests can be dominated by the hyper-LD effect and thus can provide misleading information for mapping trait genes if they are misinterpreted.

We emphasize the importance of weak and long-range correlations between SNPs in association studies. These long-range correlations can be due to genuine LD or random fluctuation or both. The magnitude of the random correlations arising by chance will decrease as the population size increases (Eq. (10)), but genuine LD between distant SNPs resulting from processes that reflect population history will persist. We speculate that more causal SNPs will be present in a larger population. If the number of causal SNPs is larger, even weaker correlations will be significant to the power of the association tests. So even in large populations, the hyper-LD effect will still be of concern.

Possible approaches to alleviating the hyper-LD effect include increasing the study population size, effectively increasing the MAFs by collapsing rare variants, using gene-set or pathway analysis, and combining information from family-based linkage or association analysis. The effectiveness of these approaches needs to be investigated in future studies.

## Competing interests

The authors declare that there are no competing interests.

## Authors’ contributions

YD supervised the study and drafted the manuscript. YD, GM, LS, and RD participated in the study design. All authors participated in the analysis of the data and revision of the manuscript. All authors read and approved the final manuscript.
